# Linking social media data with geospatial information to analyse changes in human sentiments in and along surface water environments

**DOI:** 10.1016/j.mex.2025.103603

**Published:** 2025-09-02

**Authors:** Kai-Ti Wu, Markus Venohr, Linda See, Dagmar Haase

**Affiliations:** a Humbolt Universität zu Berlin, Derpartment of Geography, Unter den Linden 6, Berlin 10099, Germany; b European Citizen Science Association, Invalidenstraße 43 10115 Berlin, Germany; c Leibniz Institute of Freshwater Ecology and Inland Fisheries, Justus-von-Liebig-Straße 7 12489 Berlin, Germany; d International Institute for Applied Systems Analysis (IIASA), Schlossplatz 1 A-2361 Laxenburg, Austria

**Keywords:** Social media data, Freshwater ecology, Large scale data, Data cleaning, Twitter, X, ecosystem services, wellbeing, sentiment analysis

## Abstract

Social media data represent a valuable source of information on human activity patterns and emotional responses in relation to natural environments. These data can provide insights into the drivers of human sentiments toward freshwater ecosystems, especially in contexts where traditional survey methods are insufficient or resource intensive. A better understanding of the relationship between human sentiments and the perceived value of freshwater environments can support the integration of public perspectives into ecosystem management and regional development. In this paper, we present a replicable method for acquiring, cleaning, and analysing geolocated Twitter data from 2011 to 2018 from Germany. The method includes multiple data cleaning and filtering steps to prepare the dataset for identifying spatial and temporal trends in sentiments and to determine the primary drivers of emotional responses to water bodies. The demonstrated workflow includes the following steps:

• Geo-located Tweets were collected via the Twitter API, then sorted, indexed, and subjected to filtering and cleaning to ensure data quality.

• Language detection and sentiment analysis using a lexicon-based method (Polyglot), suitable for limited computing power, short-text social media sentiment analysis, particularly in the context of analysing the content posted by individuals spending time in freshwater ecosystems.

• Geospatial enrichment, incorporating contextual data such as weather, population density, and other location-based variables.

## Graphical abstract

**Figure:**
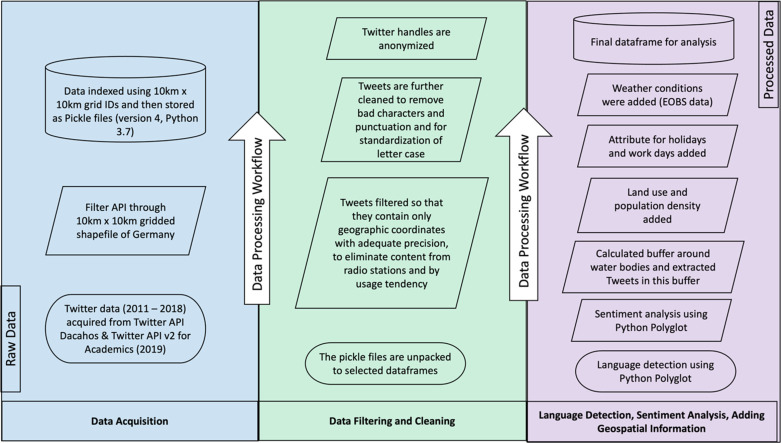

**Specifications table**

**Table:** 

**Subject area**	Environmental Science
**More specific subject area**	Environmental Science
**Name of your method**	Geo-Spatial Analysis on Freshwater Ecosystems via Twitter
**Name and reference of original method**	None
**Resource availability**	https://github.com/kaitiwu/MethodsX_LargeScaleTweets

## Background

Whilst evidence suggests that natural areas providing ecosystem services can have a positive impact on human well-being [1], there is a need for further research into the effects of freshwater ecosystems [2]. Freshwater ecosystems, including rivers, lakes, and wetlands, offer dynamic and ecologically rich environments that support a range of human-nature interactions [3]. As transitional zones between terrestrial and aquatic systems, riparian areas function as ecotones that support high biodiversity, regulate hydrological processes, and shape the sensory and experiential qualities of nearby landscapes [4]. These characteristics contribute not only to ecological services, such as flood regulation and water purification, but also to cultural ecosystem services, including recreation, aesthetic appreciation, and psychological restoration. Compared to coastal or marine ecosystems, freshwater environments are more widely distributed across inland regions and tend to be more accessible to a larger segment of the population, as is the case in Germany, where most residents, on average, have access to a freshwater body within a 2.5 km radius. Furthermore, the well-being benefits from these environments have been explored and recommended by Gascon et al [5] and Triguero-Mas et al [6].

The frequent use of freshwater ecosystems for informal recreation and nature-based activities makes them particularly relevant for examining wellbeing-related sentiments through social media expressions although only a handful of studies exist [[6], [7], [8], [9], [10], [11]]. Most research relies on the use of survey data, or they apply statistical inference to model public sentiment or nature engagement (e.g., [12,13]). Some traditional survey methods use manual surveying and self-reporting, which are collected at local or field scales and hence are limited to small geographical areas. Questionnaires or self-reported data bear risks of the so-called social desirability biases [14]. These limitations do not negate their value. Rather, they can be enhanced by combining them with data from alternative approaches. The method presented here is intended to serve as a complementary tool, particularly suited to large-scale, place-based exploration of emotional responses.

To address these limitations, we present a replicable methodological framework for collecting, storing, cleaning and processing data to study how freshwater ecosystems influence human well-being at scale. Here we interpret the “well-being benefits” from nature as indicators of subjective well-being [15], specifically its affective dimension, inferred from emotionally positive expressions in user-generated social media content related to experiences in or near natural environments. Twitter (rebranded in 2023 as X) data have long been used for opinion mining to capture human sentiments and perceptions of various social issues [[16], [17], [18]]. This allows for the capture of spontaneous, place-specific emotional responses that do not rely on recall or predefined survey categories, as well as supporting future longitudinal analyses of changes in public sentiment and engagement with natural environments over time. Users post short, timestamped messages, often accompanied by geolocation metadata. Its public and content style makes it a valuable source for studying patterns of public sentiment and engagement with specific places or themes. Hence, we selected Twitter as the social media platform to collect text-based crowdsourced data to understand the sentiment of users regarding freshwater bodies. The aim of this paper is to offer a transparent and open-source flexible pipeline with adaptable filtering logic that can be applied to other datasets and research questions in environmental perceptions, cultural ecosystem services, and human-nature interactions. By enabling researchers and policymakers to identify emotional responses to blue spaces, the method can inform interventions that promote equitable access to freshwater ecosystems, strengthen community resilience, cohesion and support sustainable development initiatives focused on public health, urban livability, and social stability. It also enables researchers to assess place-based emotional responses at scale over time and space.

## Method details

### Data acquisition

The data were obtained using the Twitter API v2 full archive, accessed on 4th March 2021 using an academic user account [19]. All geotagged data points within the study area were extracted for the period 2011 to 2018. A moving window for each 10 × 10 km<sup>2</sup> bounding box was used to ensure that all Tweets collected contained geographical information. In the context of the Twitter API, the geographical information includes “place_IDs” and “coordinates”. It was not possible to stream Tweets that contained only coordinates via the “geo” API so Tweets with “place IDs” were filtered out to extract data points with “coordinate” information.

When working with large-scale data for acquisition when the total number of data points in unknown, proper geographical indexing is important for future cross-referencing with the original data because large-scale API streaming is often subject to technical disturbances and losses of data. Hence the indexing allows us to continue data acquisition after any technical or internet accessibility disruptions. The data were selected using shapefiles implemented using the python Geopandas package so that any future analysis can be undertaken at any geographical scale. The bounding boxes are indexed and stored as Python 3.7 pickle files to match the storage space and storage speed constraints. This promotes scalability, simplifies querying and indexing, and ensures interoperability across data systems and programming languages. After acquiring all the data points, the following Tweet objects: “unique Twitter ID”, “created date”, “longitude”, “latitude” and “text” were parsed from the pickle files to produce a CSV format data frame. In total, the data set consisted of 21,656,747 rows of data from 2011 to 2018, which was then cleaned and filtered as described in the next section. Data points from bordering countries to Germany, i.e., Switzerland, France and Austria, were filtered out using a shapefile of Germany.

### Data filtering and cleaning

**Fig. 1:**
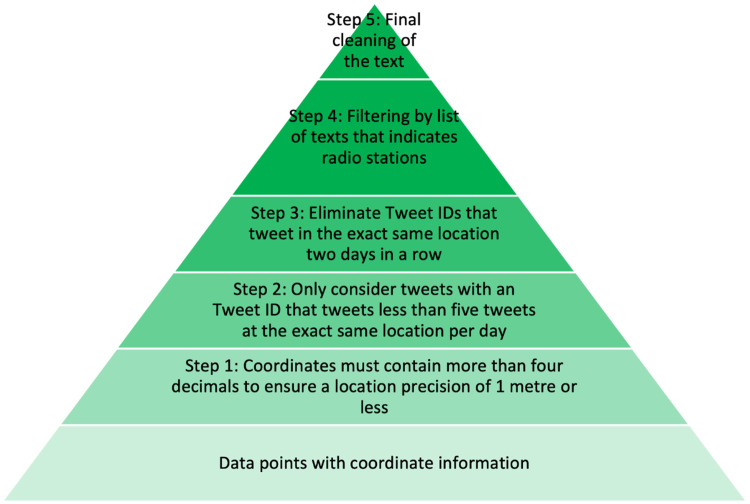
Overview of the data cleaning and filtering process.

Fig. 1 contains an overview of the steps taken in the data cleaning and filtering process. In step 1 (Fig 1), the filtered Tweets containing “coordinate” information were first filtered to ensure that they contained valid geographical coordinates with more than four decimals, which equates to a location precision of 1 m or less. In the second step, Tweets were retained if the same owner ID tweeted less than five times at the exact same location per day while in step 3, Tweet IDs with Tweets in the exact same location two days in a row were eliminated to avoid capturing users who are unlikely to be participating in surface water related recreational activities. This filtering step was introduced to reduce repetitive posting patterns at the same location on consecutive days, which can obscure broader spatiotemporal trends by overrepresenting habitual use. However, local users are a valuable population in understanding everyday nature engagement. In future studies, these parameters can and should be adjusted based on the specific research question, geographic context, or the type of behaviour being studied. In step 4, we used text filtering methods to remove any tweets containing “#playing now” in several formats, which in Germany typically indicates Tweets from German radio stations. A list of words that we used to filter out other potential radio stations can be found in the Supplementary Material (annex#1). As a result, >80 % of the data points were filtered out of the total dataset, resulting in 32,464,50 data points. Then finally in step 5, the Tweet texts were cleaned, which included unifying letter cases, and removing bad characters and punctuation.

All sensitive information such as the account handle was replaced with “Twitter Account” in order to anonymize the data. This was the final step undertaken as shown in Fig. 1. In order to protect user privacy, the shared tweets contain hashed Twitter IDs to prevent re-identification through metadata [20].

## Language detection, sentiment analysis and adding geospatial information

We then applied language detection analysis using the Python Polyglot (16.07.04) package on the data (between 2011–2018) to understand the types of languages in the database. In total, 21 different languages were detected with English (48 %), German (41 %), Turkish (4 %), Spanish (2 %) and Russian (1 %) as the top five. The Polyglot Python package assigns sentiment polarity scores at the word level based on language-specific lexicons developed from large multilingual corpora. For each tweet, Polyglot first detects the language and then applies the corresponding sentiment lexicon. Words are categorized as positive, negative, or neutral, and the overall sentiment of the post is calculated by aggregating these word-level scores. This lexicon-based method is especially well suited to short-form, emotionally expressive text typical of social media content, and it offers practical advantages in low-resources computational environments [21,22].

We also used the language packages from Polyglot to analyse the sentiment value of the Tweet texts, which ranged from −1 to +1. We grouped the Tweets with values between −0.4 to −1.0 as negative tweets, −0.3 to 0.3 as neutral tweets, and 0.4 to 1 as positive Tweets. 18.5 % of Tweets were found to be positive, 12.4 % of the Tweets were detected as negative, while the majority (69 % of Tweets) had neutral sentiments.

Finally, we added location specific information to the Tweets. This included information on land cover and land use from the LandBedeckungsModel (Bundesamt für Kartographie und Geodäsie (BKG), 2019) and Population density data (Zensus 2011 Bevölkerungsraster) (Statistisches Bundesamt (Destatis), 2011) processed into 100 m raster resolution using ArcGIS10.1. The workday and holiday data were acquired for the period 2011 to 2018 by collecting German state-wise administrative calendar information. We then assigned a binary value to each date, expressing the holiday or workday information as binary values. This was necessary because different states in Germany have different holidays in respective years. The distance to water was calculated based on the processed surface water layer of polygons from OpenStreetMap and the spatial datatsets from the WISE Water Framework Directive database. We used the point to polygon tool from ArcGIS Pro 2.1. The weather condition data were based on the 24.0e European Climate Assessment & dataset (E-OBS version 24.0e**.** *European Climate Assessment & Dataset (ECA&D)* (https://www.ecad.eu/download/ensembles/download.php), which includes solar radiation, precipitation and temperature data. We tagged them using Python 3.9 by accessing grid information through the netCDF4 1.6.5 package. The result is a data frame containing Tweet information and geotagged data.

## Method validation

We tested the validity of the algorithm-generated sentiment values with 15 researchers from the Aquatag project. Each participant evaluated 25 samples across five sentiment classes (−1, −0.5, 0, 0.5, 1) and received 50 identical tweets to rate. The results showed an overall accuracy of 68 % across all values, and an 85 % agreement for polarized sentiments (−1 and 1).

## Limitations and discussion

Social media data provide large databases, but they also come with limitations. We have characterised the limitations of this method as follows:

1. Accessibility: The rules to gain access to Tweets for research vary and are not consistent [23]. High costs may apply for accessing a large quantity of data so this constraint may limit certain types of research that can be undertaken through the platform, depending on the budget of the researcher. Since the data collection policies of Twitter/X have changed over time, this may affect longitudinal studies.
2. Quality: Geospatial gaps may occur when users opt out from sharing location data, making geographic analysis incomplete. Texts from social media may contain slang, appear unstructured or have multiple meanings, which may limit the ability to interpret them accurately. Demographic information is not available through social media data, and with personal preferences in place, one might not get a representative reflection of the general population.
3. User consistency: As social media platforms evolve, changes in user demographics, data availability, and geotagging behaviour may introduce temporal biases. Future applications of this method should consider these shifts and apply appropriate validation strategies when comparing data across time periods.

A substantial share of the posts in our dataset were classified as neutral, which could be typical of short-form, descriptive social media content that may not contain emotional language. While our method currently focuses on polarity-based sentiment analysis, future extensions could explore the neutral category using topic modelling, key word extraction, or discourse analysis. These approaches may uncover latent patterns in language that reflect subtle or implicit forms of engagement with natural environments, offering additional depth to studies of human-nature interactions.

While our study relied on the Polyglot sentiment lexicon (last updated in 2016), its temporal alignment with our dataset (2011–2018) makes it a reasonable choice for the period under investigation. However, we fully acknowledge that lexicon-based methods can benefit from ongoing linguistic and cultural updates, particularly in the context of social media data where sentiment expressions evolve rapidly. Future research could explore more recent models, such as MultiLexScaled [24] or adaptive multillingual lexicon-based approaches such as Lex2Sent [25], which also adapts hybrid methods including machine learning approaches.

The outputs presented in this paper are not intended as definitive results, but rather as illustrations of how the method can be applied to large-scale, multilingual, and geotagged social media data. These examples demonstrate the feasibility of detecting sentiment patterns across space and time, which may be useful for exploring associations between public mood, nature engagement, and environmental access. While we do not offer full empirical analysis here, the visualizations and sentiment distributions illustrate how this workflow can support future research into temporal trends, regional disparities, or the emotional relevance of specific landscape types. We recognize that, in their current form, these outputs are limited in scope and not statistically validated. However, the structure presented invites readers to formulate specific research questions and explore them further, supported by a transparent and replicable method. This approach is particularly relevant for interdisciplinary fields such as environmental psychology, urban planning, and subjective well-being research.

## Concluding remarks

In summary, the method offers a scalable approach for capturing public sentiment in relation to natural environments across spatial and temporal contexts in addition to advancing digital methods in environmental psychology, recreational behavior, and potential links to subjective well-being research at spatial and temporal scales. While it does not replace traditional survey methods, it complements them by offering large scale spatiotemporal information and provides additional perspectives regarding public sentiments, and can be used to develop policies that promote equitable access to freshwater ecosystems.

## Ethics statements

The authors have carefully reviewed the EU data privacy regulation (GDPR) and checked for updates until before the time of submission. The authors have complied with the usage rules as set out on the platform X. All data retrieved from X have been carefully handled concerning data storage, anonymization and processing. The data presented in this paper were obtained using X’s services strictly adhering to their Terms of Service. As per X’s copyright policy, tweets may be owned by various entities. However, we have safeguarded the privacy rights of individuals in this dataset by removing each user's identity from the tweets and comments.

## CRediT authorship contribution statement

**Kai-Ti Wu:** Conceptualization, Methodology, Software, Formal analysis, Data curation, Software, Visualization, Writing – original draft, Writing – review & editing. **Markus Venohr:** Funding acquisition, Supervision, Conceptualization, Project administration, Methodology, Writing – review & editing, Validation. **Linda See:** Supervision, Writing – review & editing, Validation. **Dagmar Haase:** Supervision, Writing – review & editing.

## Declaration of competing interest

The authors declare that they have no known competing financial interests or personal relationships that could have appeared to influence the work reported in this paper.

## Data Availability

https://github.com/kaitiwu/MethodsX_LargeScaleTweets.

## Appendix Supplementary materials

Image, application 1
