# Supplementary material for: Linking social media data with geospatial information to analyse changes in human sentiments in and along surface water environments
Source: MethodsX. 2025 Sep 2;15:103603. doi: 10.1016/j.mex.2025.103603 (PMC12450559; doi:10.1016/j.mex.2025.103603)
Supplement: Supplementary file 1 [file mmc1.docx]

1. #NowPlaying
2. #nowplaying
3. #NOWPLAYING
4. #Nowplaying
5. #nowPlaying
6. #PlayingNow
7. #playingnow
8. #PLAYINGNOW
9. #Playingnow
10. #playingNow
11. #OnAirNow
12. #onairnow
13. #ONAIRNOW
14. #Onairnow
15. #onAirNow
16. #NowPlayingOnRadio
17. #nowplayingonradio
18. #NOWPLAYINGONRADIO
19. #Nowplayingonradio
20. #nowPlayingOnRadio
